# Beyond the Sensorimotor Plasticity: Cognitive Expansion of Prism Adaptation in Healthy Individuals

**DOI:** 10.3389/fpsyg.2015.01979

**Published:** 2016-01-05

**Authors:** Carine Michel

**Affiliations:** ^1^Unité de Formation et de Recherche en Sciences et Techniques des Activités Physiques et Sportives, Campus Universitaire, Université de BourgogneDijon, France; ^2^INSERM, U 1093, Cognition, Action et Plasticité SensorimotriceDijon, France

**Keywords:** prism adaptation, cognition, after-effects, pseudoneglect, neglect

## Abstract

Sensorimotor plasticity allows us to maintain an efficient motor behavior in reaction to environmental changes. One of the classical models for the study of sensorimotor plasticity is prism adaptation. It consists of pointing to visual targets while wearing prismatic lenses that shift the visual field laterally. The conditions of the development of the plasticity and the sensorimotor after-effects have been extensively studied for more than a century. However, the interest taken in this phenomenon was considerably increased since the demonstration of neglect rehabilitation following prism adaptation by Rossetti et al. (1998). Mirror effects, i.e., simulation of neglect in healthy individuals, were observed for the first time by Colent et al. (2000). The present review focuses on the expansion of prism adaptation to cognitive functions in healthy individuals during the last 15 years. Cognitive after-effects have been shown in numerous tasks even in those that are not intrinsically spatial in nature. Altogether, these results suggest the existence of a strong link between low-level sensorimotor plasticity and high-level cognitive functions and raise important questions about the mechanisms involved in producing unexpected cognitive effects following prism adaptation. Implications for the functional mechanisms and neuroanatomical network of prism adaptation are discussed to explain how sensorimotor plasticity may affect cognitive processes.

## Introduction

Sensorimotor plasticity allows producing an appropriate motor response in reaction to environmental changes or bodily evolution during the life. Prism adaptation is one of the oldest paradigms to study sensorimotor plasticity. It consists of pointing to visual targets while wearing prismatic lenses that shift the visual field laterally. The pointing errors made in the direction of the optical shift are gradually corrected. After prism removal, the pointing movements are shifted in the direction opposite to the optical deviation. The entire process can be explained by proprioceptive, visual, and motor control changes (e.g., Kornheiser, 1976). Prism adaptation has been described for more than one century (e.g., Stratton, 1896) but the interest taken in this procedure was considerably increased since the publication of the article of Rossetti et al. (1998) showing the therapeutic impact of prism adaptation in neglect rehabilitation (Newport and Schenk, 2012). Neglect is described as a failure to report, respond, or orient to novel or meaningful stimuli presented to the side opposite to a brain lesion usually in the right hemisphere (e.g., Heilman et al., 1993). In their comprehensive review, Jacquin-Courtois et al. (2013) set out the therapeutic interest of prism adaptation on a broad range of clinical and experimental measures beyond the framework of sensorimotor coordination. The present revue focuses on the cognitive after-effects of prism adaptation in healthy individuals and presents this procedure as a robust tool for simulating neglect in normals. In the light of the recent results, it offers potential insights into the understanding of the expansion of low-level sensorimotor processes to cognitive functions.

### Cognitive After-Effects of Prism Adaptation in Space Representation

The first demonstration of prism-induced cognitive after-effects in space representation (mental image of the space mapped across the brain) in healthy individuals was shown Colent et al. (2000). Line bisection task is an invaluable tool to assess space representation. In the manual version, subjects place a mark at the center of a horizontal line. In the perceptual judgment version (landmark test), they are requested to judge whether a line has been transected to the left or the right of its true center. Performance in healthy subjects is characterized by a leftward pseudoneglect bias due to a mental over-representation of the left part of space and an under-representation of the right part of space (e.g., McCourt and Jewell, 1999). Neglect patients show a rightward bias because they exhibit a mental under-representation of the left part of space and an over-representation of the right part of space (e.g., Halligan, 1995). When adaptation was developed by visuo-manual pointings with the right dominant hand during prism exposure (20 min under 15° leftward or rightward deviation), only adaptation to a leftward optical deviation produced representational after-effects. They appeared to be a faithful qualitative simulation of neglect, i.e., a rightward bias in perceptual line bisection (Colent et al., 2000). Complementary studies confirmed the effects of adaptation on both manual and perceptual bisection tasks (Michel et al., 2003a; Nijboer et al., 2010; Striemer and Danckert, 2010; Fortis et al., 2011; Michel and Cruz, 2015) and showed that they lasted for at least half an hour (Schintu et al., 2014). The occurrence of cognitive after-effects in healthy individuals seems to depend on the baseline expression of pseudoneglect. A greater leftward magnitude at baseline is associated with greater rightward after-effects (Goedert et al., 2010; Herlihey et al., 2012). Therefore, individuals with leftward bias due to right hemisphere dominance (e.g., Fink et al., 2001) are particularly sensitive to adaptation to leftward optical deviation that may act on right hemisphere functioning (see paragraph 5). This could also be the reason why experimental conditions involving the right hemisphere, as left-sided location of the line, favor the occurrence of cognitive after-effects (Michel et al., 2003a) and why no after-effect was observed in manual line bisection in the absence of baseline pseudoneglect (Colent et al., 2000).

Cross-modal after-effects of prism adaptation are observed on haptic tasks where participants are required to locate the center of a haptically explored circle or a visually perceived circle. Prism adaptation induced a rightward shift of performance similar to the bias shown in neglect patients (McIntosh et al., 2002; Girardi et al., 2004). Cognitive after-effects also extend to extrapersonal representation, i.e., beyond the immediate region (arm reach) within which visuomotor adaptation takes place. They were shown in the landmark task (Berberovic and Mattingley, 2003) and in goal-oriented locomotion (Michel et al., 2008). They are similar to neglect-like locomotor bias (Robertson et al., 1994; Berti et al., 2002). Cognitive after-effects even occur in mental scales. The mental number line is thought to have a left-to-right organization whereby low and high numbers are represented along a spatial continuum from left to right (Dehaene et al., 1993). As a result, when judging the distance between two numbers, without using arithmetic, normal subjects misbisect the mental distance toward the smaller number (i.e., to the left) (Longo and Lourenco, 2007; Loftus et al., 2009a). Adaptation to a leftward optical deviation was responsible for a shift in bisection toward the high numbers (i.e., to the right) (Loftus et al., 2008) that could be compared to a mild neglect-like behavior (Vuilleumier et al., 2004; Zorzi et al., 2002, 2006). The mental alphabetic line has also a left-to-right organization with early letters on the left side and later letters on the right side of space (Gevers et al., 2003; Zorzi et al., 2006). Normal subjects misbisect the mental distance toward early letters (i.e., to the left) (Zorzi et al., 2006; Nicholls and Loftus, 2007). Adaptation to a leftward optical deviation produces a shift in bisection toward the later letters (i.e., to the right) (Nicholls et al., 2008) that could be compared to a mild neglect-like behavior (Gevers et al., 2003; Zorzi et al., 2006). Cognitive after-effects also concern body representation. The analysis of the center of pressure (point of application of the ground reaction force vector) is a useful but indirect tool to assess the internal model of the body (Gurfinkel and Levick, 1991). Adaptation to a leftward optical deviation produced a rightward shift of the center of pressure in an eyes closed condition which correlated negatively with a counterclockwise estimation of the visual vertical (encoded within an egocentric frame of reference) (Michel et al., 2003b). These results on posture and subjective visual vertical are similar to mild symptoms following right brain lesion and more particularly neglect manifestations (Bohannon et al., 1986; Brandt et al., 1994; Rode et al., 1997, 1998; Kerkhoff, 1999; Tilikete et al., 2001). Altogether, prism adaptation affects a supramodal level of space representation in both explicit and non-explicit spatial tasks.

We could mention here that prism adaptation affects also spatial remapping that enables the construction of a stable representation of the visual environment despite constantly changing retinal images. Prism adaptation to a leftward optical deviation induces impairment in spatial remapping for left visual field targets in the double-step saccade paradigm (Bultitude et al., 2013c). These after-effects could be viewed as mild neglect behavior (Pisella et al., 2011).

### After-Effects of Prism Adaptation in Attention and Hierarchical Processing

Because space representation depends on orientation of attention (e.g., Milner et al., 1992), the rightward representational after-effects described above could be partly explained by a redistribution of spatial attention to the detriment of the left space. Prism adaptation affects covert attention (Striemer et al., 2006) and has lateralised effects on spatial attention. Judgment of luminance (grayscales task) requires a forced choice judgment between two mirror-reversed luminance gradients. Participants usually select the stimulus that is darker on the left despite the fact that both stimuli are equiluminant (Nicholls et al., 1999; Okubo and Nicholls, 2006). This leftward bias is reversed following adaptation to a leftward optical deviation (Loftus et al., 2009b) mimicking neglect-like behavior in grayscales (Sarri et al., 2011).

The influence of prism adaptation extends also to hierarchical processing as first demonstrated by Bultitude and Woods (2010). When healthy participants are presented with figures in which small letters are arranged to form a large letter (Navon, 1977), they are faster to identify the global-level than the local-level information, and have difficulty ignoring global information when identifying the local level. After adaptation to a leftward optical deviation, there was a significant reduction in global interference similar to the processing bias demonstrated in patients with right temporo-parietal junction lesions (Bultitude et al., 2009). Reed and Dassonville (2014) demonstrated that adaptation to a leftward optical deviation increased the susceptibility to a subset of visual illusions known to be driven by local contextual processing. However, adaptation failed to influence performance in the composite face task that is supposed to evaluate the automatic global-level processing of faces (Bultitude et al., 2013a). Negative results were also observed on spatial attention in space-based or object-based attention (Bultitude et al., 2013b), in a temporal order judgment task (Berberovic et al., 2004), in saccade latencies or antisaccade errors (Nijboer et al., 2010) and in visual search (Morris et al., 2004; Saevarsson et al., 2009). Even if a lack of sensitivity cannot be excluded for several tasks, these negative results could be explained by the absence of pseudoneglect behavior in baseline performance. It has been proposed that any aspects of performance that have been altered by prism adaptation are ones for which the behavior is already biased toward pseudoneglect (Nijboer et al., 2010; Bultitude et al., 2013b) (see Paragraph 1). Therefore, the influence of prism adaptation could be viewed as reducing pseudoneglect or inversing pseudoneglect to produce mild neglect.

### Why aren’t Cognitive After-Effects Explained by Sensorimotor After-Effects?

The occurrence of cognitive after-effects is even more interesting when considering that the development of prism adaptation relies on active motor behavior and that cognitive after-effects cannot be explained in terms of sensorimotor after-effects. Several arguments could be presented here. (1) If cognitive after-effects were explained by sensorimotor after-effects they would be mainly observed in tasks involving visuo-manual coordination. On the contrary, they are shown in tasks requiring verbal responses (Berberovic and Mattingley, 2003; Michel et al., 2003a; Loftus et al., 2008, 2009b; Nicholls et al., 2008; Nijboer et al., 2010; Schintu et al., 2014). (2) If cognitive after-effects were explained by sensorimotor after-effects the amplitude of the bias in bisection task would be independent of the spatial location because sensorimotor after-effects generalize homogeneously over space (Bedford, 1989). The bias would have also the same amplitude irrespective of the length of the line because relative position coding is not altered by wedge prisms (Redding and Wallace, 1996). On the contrary, line bisection bias is greater for left-sided locations than for right sided-locations and it increases with the line length (Michel et al., 2003a). (3) If cognitive after-effects were explained by sensorimotor after-effects they would be symmetric in amplitude following exposure to left and right optical deviations (Cohen, 1967; Loftus et al., 2008; Michel et al., 2003b, 2008; Schintu et al., 2014). On the contrary, cognitive after-effects on pseudoneglect behavior occur only following adaptation to a leftward optical deviation (Colent et al., 2000; Michel et al., 2003b; Loftus et al., 2008, 2009b; Nicholls et al., 2008; Bultitude and Woods, 2010; Goedert et al., 2010) (4) If cognitive after-effects result from sensorimotor after-effects they could be partly explained by visual after-effects. Prism adaptation is responsible for visual displacement of the gaze in the direction of the optical deviation during and after prism exposure (e.g., Wallace et al., 1973) due to eye muscle potentiation (Ebenholtz, 1974) and visual recalibration (Craske, 1975; Craske and Crawshaw, 1975). Because line bisection performance is biased toward the start location of the scanning direction (Brodie and Pettigrew, 1996; Chokron et al., 1998) a leftward bias could be expected after adaptation to a leftward optical deviation. On the contrary, a rightward bias is observed in line bisection (e.g., Colent et al., 2000; Striemer and Danckert, 2010) and in ocular exploration (Ferber and Murray, 2005) showing a reorganization of the visual functions that cannot be explained by visual after-effects. (5) If cognitive after-effects were explained by sensorimotor after-effects there would be a correlation between the amplitude of sensorimotor and cognitive after-effects. On the contrary, all studies analyzing the link between sensorimotor and cognitive after-effects showed no correlation (Berberovic and Mattingley, 2003; Girardi et al., 2004; Fortis et al., 2011; Herlihey et al., 2012; Guinet and Michel, 2013; Schintu et al., 2014).

Even if cognitive after-effects cannot be directly explained by sensorimotor after-effects, their occurrence strictly depends on the development of adaptation (spatial realignment) which needs active pointing movements during prism exposure (Michel et al., 2003a). Furthermore the spatial realignment must be strong enough (by using at least 10° optical deviation) to observe cognitive after-effects (Michel and Cruz, 2015). Nevertheless, the attempt to increase cognitive after-effects by combining neck muscle vibration with prism adaptation to increase the misperception of the target in the direction of the prismatic shift is unfruitful in healthy individuals (Guinet and Michel, 2013).

### Sensorimotor and Cognitive After-Effects: Where is the Boundary?

The term ‘cognitive’ refers to the fact that effects take place beyond the usual framework of compensatory sensorimotor after-effects and involves mental abilities. Cognitive after-effects are mainly assessed by ‘paper-pencil’ tests or need verbal responses as manual line bisection or mental number bisection, respectively. They result from higher cognitive processes involved in judgment and comparison. In contrast, sensorimotor after-effects are exclusively shown in tasks assessing sensorimotor coordination as visuo-manual open-loop pointing. They result from adaptive changes in perception and motor command. Nevertheless, under certain circumstances, when a motor response is required for a cognitive task, both types of after-effects can coexist. This could be the case for the manual line bisection but the use of slow movements under visual guidance to set the bisection mark abolishes any sensorimotor influence (Redding and Wallace, 1996). The absence of correlation between cognitive and sensorimotor after-effects indicates that sensorimotor after-effects do not influence cognitive responses (see Paragraph 3). On the contrary, goal-oriented locomotion to a memorized visual target allows the coexistence of both types of after-effects because it involves memori*z*ed representation of a target in space for a few seconds (before and during the displacement) which favors the appearance of representational after-effects in far space (Michel et al., 2008). Moreover, as mentioned in Paragraph 3, the optical deviation used to produce after-effects is to consider. Cognitive after-effects are asymmetric whereas sensorimotor after-effects are symmetric.

### From Pseudoneglect to Neglect-Like Behavior: How is it Possible?

Attempts to produce neglect in normals are based on the specialization of the human right hemisphere for visuo-spatial functions (e.g., Benton and Tranel, 1993). Interhemispheric changes to the detriment of the right hemisphere (or in favor of the left hemisphere) produce a mild neglect-like behavior or reduce pseudoneglect. For clarity and conciseness, only examples from line bisection studies are presented here. Leftward pseudoneglect bias in line bisection decreases when the right hand (versus left hand) is used (Scarisbrick et al., 1987; Fukatsu et al., 1990; Brodie and Pettigrew, 1996; Jewell and McCourt, 2000), when lines are located in right hemispace (Reuter-Lorenz et al., 1990; Luh, 1995; McCourt and Jewell, 1999) or when attention is oriented to the right extremity of the line (Milner et al., 1992; Harvey et al., 2000). Aging is also characterized by hemispheric changes. Faster aging of the right hemisphere (Meudell and Greenhalgh, 1987; Robinson and Kertzman, 1990) or reduced hemispheric asymmetry with aging (Dolcos et al., 2002; Reuter-Lorenz, 2002; Cabeza et al., 2004), may be responsible for a rightward bias in line bisection (Fujii et al., 1995; Schmitz and Peigneux, 2011; Benwell et al., 2014). Direct modulation of the cerebral activity by using transcranial magnetic stimulation (TMS) or transcranial direct current stimulation (tDCS) produces also neglect-like symptoms (Sack, 2010). The use of repetitive TMS over right frontal and right posterior parietal cortices produces a rightward neglect-like bias in line bisection (Fierro et al., 2000, 2001; Brighina et al., 2002; Ellison et al., 2004; Bjoertomt et al., 2009), in mental number line (Göbel et al., 2006) or in visual target detection (Thut et al., 2005; Muggleton et al., 2006). When right cathodal (hyperpolarization) and left anodal (depolarization) tDCS were simultaneously applied over homolog posterior parietal cortices or when only right cathodal tDCS was used, a rightward bias in landmark task was shown (Giglia et al., 2011; Benwell et al., 2015). Interestingly enough, when neglect-like bias was produced during TMS over the right parietal cortex, functional imaging studies showed a decreased activity within the site of stimulation and in interconnected right hemisphere structures and even enhanced BOLD signal in the left parietal and visual cortices (Sack et al., 2007; Heinen et al., 2011; Ricci et al., 2012).

Therefore, interhemispheric imbalance to the detriment of the right parietal cortex may explain prism-induced cognitive after-effects (Michel, 2006). Studies in brain damaged patients and healthy individuals underline the involvement of the parieto-cerebellar network in the development of adaptation (Clower et al., 1996; Pisella et al., 2005; Luauté et al., 2006, 2009; Danckert et al., 2008; Chapman et al., 2010; Crottaz-Herbette et al., 2014). The temporal cortex, involved during realignment, might also account for some of cognitive after-effects (Luauté et al., 2009). Otherwise, the hand used during prism exposure may have a potential influence on the hemispheric imbalance following adaptation. Except two studies (Michel et al., 2008; Reed and Dassonville, 2014) using both hands for visuo-manual pointings during prism exposure, the right dominant hand (involving the left hemisphere) is always used.

## Conclusion

Prism adaptation is undoubtedly a fascinating phenomenon that urges us to revisit our conception of sensorimotor plasticity and questions us on the reciprocal relations between cognition and action. Prism adaptation is a powerful non-invasive method for neglect rehabilitation (e.g., Rossetti and Rode, 2002; Rode et al., 2015) that is able to mirror neglect in normal. Nevertheless the neural substrate of after-effects during a cognitive task following prism adaptation to a leftward optical deviation (conditions known to express neglect-like behavior) has not yet been studied in healthy individuals. Furthermore, the nature of the after-effects needs to be further investigated because it does not limit to sensorimotor and cognitive domains (e.g., Sumitani et al., 2007).

## Conflict of Interest Statement

The author declares that the research was conducted in the absence of any commercial or financial relationships that could be construed as a potential conflict of interest.
